# Main characteristics of patients of primary health care services in Brazil

**DOI:** 10.11606/S1518-8787.2017051007070

**Published:** 2017-09-22

**Authors:** Ione Aquemi Guibu, José Cássio de Moraes, Augusto Afonso Guerra, Ediná Alves Costa, Francisco de Assis Acurcio, Karen Sarmento Costa, Margô Gomes de Oliveira Karnikowski, Orlando Mario Soeiro, Silvana Nair Leite, Juliana Álvares

**Affiliations:** I Departamento de Saúde Coletiva. Faculdade de Ciências Médicas da Santa Casa de São Paulo. São Paulo, SP, Brasil; II Departamento de Farmácia Social. Faculdade de Farmácia. Universidade Federal de Minas Gerais. Belo Horizonte, MG, Brasil; III Instituto de Saúde Coletiva. Universidade Federal da Bahia. Salvador, BA, Brasil; IV Núcleo de Estudos de Políticas Públicas. Universidade Estadual de Campinas. Campinas, SP, Brasil; V Programa de Pós-Graduação em Saúde Coletiva. Departamento de Saúde Coletiva. Faculdade de Ciências Médicas. Universidade Estadual de Campinas. Campinas, SP, Brasil; VI Programa de Pós-Graduação em Epidemiologia. Faculdade de Medicina. Universidade Federal do Rio Grande do Sul. Porto Alegre, RS, Brasil; VII Faculdade de Ceilândia. Universidade de Brasília. Brasília, DF, Brasil; VIII Faculdade de Ciências Farmacêuticas. Pontifícia Universidade Católica de Campinas. Campinas, SP, Brasil; IX Departamento de Ciências Farmacêuticas. Universidade Federal de Santa Catarina. Florianópolis, SC, Brasil

**Keywords:** Pharmaceutical Services, utilization, Patients, Health Profile, Socioeconomic Factors, Primary Health Care, Health Services Research, Unified Health System, Assistência Farmacêutica, utilização, Pacientes, Perfil de Saúde, Fatores Socioeconômicos, Atenção Primária à Saúde, Pesquisa sobre Serviços de Saúde, Sistema Único de Saúde

## Abstract

**OBJECTIVE:**

To characterize patients of primary health care services according to demographic and socioeconomic aspects, habits and lifestyle, health condition, and demand for health services and medicines.

**METHODS:**

This study is part of the *Pesquisa Nacional sobre Acesso, Utilização e Promoção do Uso Racional de Medicamentos – Serviços* (PNAUM – National Survey on Access, Use and Promotion of Rational Use of Medicines – Services), a cross-sectional study carried out between 2014 and 2015. Interviews were conducted with patients over the age of 17 years, with a standardized questionnaire, in primary health care services of a representative sample of cities, stratified by regions of Brazil. The analysis was performed for complex samples and weighted according to the population size of each region.

**RESULTS:**

A total of 8,676 patients were interviewed, being 75.8% women, most of them aged from 18 to 39 years; 24.2% men, most of them aged from 40 to 59 years; 53.7% with elementary school; 50.5% reported to be of mixed race ethnicity, 39.7%, white, and 7.8%, black. Half of patients were classified as class C and 24.8% received the *Bolsa Familia* benefit. Only 9.8% had health insurance, with higher proportion in the South and lower in the North and Midwest. The proportion of men who consumed alcohol was higher than among women, as well as smokers. The self-assessment of health showed that 57% believed it to be very good or good, with lower proportion in the Northeast. The prevalence of chronic diseases/conditions, such as hypertension (38.6%), dyslipidemia (22.7%), arthritis/rheumatism (19.4%), depression (18.5%), diabetes (13.6%), and others are higher in these patients them among the general population. Medicines were predominantly sought in the health care service or in pharmacies of the Brazilian Unified Health System.

**CONCLUSIONS:**

It was possible to characterize the profile of patients of Primary Health Care, but the originality of the research and its national scope hinders the comparison of results with official data or other articles.

## RESUMO

**OBJETIVO:**

Caracterizar os usuários dos serviços de atenção primária à Saúde segundo aspectos demográficos, socioeconômicos, hábitos e estilo de vida, condições de saúde referidas e demanda aos serviços de saúde e medicamentos.

**MÉTODOS:**

Foram utilizados dados da Pesquisa Nacional sobre Acesso, Utilização e Promoção do Uso Racional de Medicamentos – Serviços, um estudo transversal, exploratório, de natureza avaliativa, realizado entre 2014 e 2015. As entrevistas foram realizadas com usuários maiores de 17 anos, com questionário padronizado, em serviços de atenção primária de amostra representativa de municípios, estratificada pelas regiões do Brasil. A análise realizada foi para amostras complexas e ponderada de acordo com o tamanho da população de cada região.

**RESULTADOS:**

No total foram entrevistados 8.676 usuários, sendo 75,8% mulheres, a maioria de 18 a 39 anos de idade; 24,2% homens, e entre estes, a maioria de 40 a 59 anos; 53,7% com ensino fundamental; 50,5% se autodeclararam pardo, 39,7% branco e 7,8% preto. Metade dos usuários foi classificada como classe C e 24,8% recebia bolsa-família. Apenas 9,8% possuía plano de saúde, com maior proporção no Sul e menor no Norte e no Centro-Oeste. A proporção de homens que consumia bebida alcóolica foi maior que entre mulheres, assim como os fumantes. A autoavaliação de saúde mostrou que 57% acreditavam ser muito boa ou boa, com menor proporção no Nordeste. As prevalências de doenças/condições crônicas como hipertensão (38,6%), dislipidemia (22,7%), artrites/reumatismo (19,4%), depressão (18,5%), diabetes (13,6%) e outras são maiores nesses usuários que na população geral. Os medicamentos eram predominantemente procurados no próprio serviço ou em farmácias do Sistema Único de Saúde.

**CONCLUSÕES:**

Foi possível caracterizar o perfil dos usuários da Atenção Básica, mas o ineditismo da pesquisa e sua abrangência nacional dificulta a comparação dos resultados com dados oficiais ou de outros artigos.

## INTRODUCTION

Primary Health Care (PHC) has been developing for decades, getting different denominations and approaches and, with the implementation of the Brazilian Unified Health System (SUS), it is considered the entrance to the use of health care services for the population, at all levels of complexity^10^. Marsiglia^10^ analyzed the principles and the evolution of the concepts and practices at an international scope and within Brazil that “primary care” has been developed for its current denomination. PHC is explicit in the national health policy, with a central role for SUS to provide access to a comprehensive and quality health care for the population^12^.

The Brazilian Ministry of Health, by Ordinance no. 3,916/GM of October 30, 1998, established the National Drug Policy to ensure the required safety, efficacy, and quality of these products, the promotion of rational use, and the access of the population to those considered essential (*Relação Nacional de Medicamentos Essenciais* (Rename – National List of Essential Medicines))^11^. It also established other guidelines, such as the reorientation of Pharmaceutical Services, sanitary regulations for medicines, promotion and production of medicines^11^.

Currently, within the structure of the Department for Pharmaceutical Services and Strategic Health Supplies of the Brazilian Ministry of Health, there is the Basic Component of Pharmaceutical Services, responsible for the medicine policy for PHC^a^. This supply is funded by the three levels of government (federal, state, and municipal) and should be applied in the costing of medicines for prevalent and priority diseases of the Primary Health Care, present in the Rename in force^a^.

Knowing the general characteristics of PHC patients and the characteristics of the respective coverage areas is essential for the planning and management of health care services and actions. The Brazilian Ministry of Health implemented, in 1998, the *Sistema de Informação da Atenção Básica* (SIAB – Information System of Primary Health Care) – replacing the *Sistema de Informação do Programa de Agentes Comunitários de Saúde* (SIPACS – Information System of the Program of Community Health Agents) –, for monitoring the actions and results of the activities carried out by the teams of *Programa Saúde da Família* (PSF – Family Health Program)^15^.

SIAB was developed as a management tool of the *Sistemas Locais de Saúde* (SILOS – Local Health Systems) and its formulation incorporated concepts such as territory, problem, and sanitary responsibility. It is an important system for obtaining official information on PHC units^15^.

SIAB has registrations of families, housing and sanitation conditions, health status, production and composition of health teams, and this database is available on the internet^b^.

However, it is not possible to verify, among the total people registered, how many actually use the units. We can only obtain data on pregnant women, children, and some diseases, such as hypertension, diabetes, and tuberculosis.

The *Pesquisa Nacional sobre Acesso, Utilização e Promoção do Uso Racional de Medicamentos* (PNAUM – National Survey on Access, Use and Promotion of Rational Use of Medicines) had two national coverage strategies: household survey and services survey. The main goal of the services component was to characterize the organization of pharmaceutical services in the primary health care provided by SUS – for promoting the access and rational use of medicines –, as well as to identify and discuss the factors that interfere in the consolidation of pharmaceutical services in the cities.

The originality and the large amount of information surveyed in this research will provide in-depth analyses on aspects related to pharmaceutical services in PHC and will be approached in specific articles of this supplement.

This study aimed to characterize patients of primary health care services according to demographic and socioeconomic aspects, habits and lifestyle, health conditions, and demand for health services and medicines.

## METHODS

The methodology of PNAUM – Services, as well as the sampling process, are described in detail in this supplement by Álvares et al.^1^

PNAUM is a cross-sectional study, consisting of a survey of information in a representative sample of primary health care services, in cities of the Brazilian regions. Several study populations were considered in the sampling plan, with samples stratified by regions, which constitute the study domains^1^. Face-to-face interviews were held with patients, doctors, and those responsible for the delivery of medicines in primary health care services of SUS, in addition to observation of pharmaceutical services facilities and telephone interviews with those responsible for these services and with municipal health care managers. Data were collected from July to December 2014.

Sampling was carried out in three steps: cities, services, and patients. Initially, cities were drawn from each geographic region. In each region, 120 cities were drawn, including all the capitals of the states and cities with different population sizes. The *Unidades Básicas de Saúde* (UBS – Basic Health Units) of 60 of the 120 cities constituted the population of services. In all five regions of Brazil, 1,541 UBS were drawn. In the third step, six to seven patients per UBS were drawn.

The draw of patients in each service could not be performed from patient listings, as would be expected in probability sampling. We established criteria for the selection of patients that did not allow interviewers to choose which patients would compose the sample, approximating at the most the selection to a random drawing.

Interviewers were instructed to verify the number of interviews to be conducted on each service and calculate the number of days required to complete these interviews. In the health care service, after obtaining the consent of the coordinator for the data collection required for the research, we surveyed the names of all consultant doctors in each day that the interviewer remained in the service. The names were listed alphabetically in the planning worksheet of the draw and the interviews were distributed to the doctors of the worksheet, according to alphabetical order. After this step, we identified the patients that would be interviewed and the interview began by the reverse order of the doctor’s schedule, i.e., from the last scheduled patient and present on the day of the interview to the first ones.

The interviews were conducted by trained interviewers, by a structured questionnaire for patients with more than 17 years of age and who were present in the unit at the time of the interview. The questionnaire contained questions regarding demographic and socioeconomic characterizations, chronic disease/conditions, demand for health services, habits and lifestyle, and use of at least one medicine. One could fill in the details of up to 20 medicines. The time spent for this interview varied depending on the number of comorbidities and the amount of medicines used. We used the economic classification of the *Associação Brasileira de Empresas de Pesquisas* (ABEP– Brazilian Association of Research Enterprises), 2014 version^c^.

Data were processed in the SPSS program, version 17, in the analysis module for complex samples. Results were weighted according to the population size of each region. The significance of the difference between groups was determined by overlapping or not the 95% confidence intervals (95%CI).

All participants signed the informed consent form. PNAUM was approved by the National Research Ethics Committee of the National Health Council, by Opinion no. 398,131/2013.

## RESULTS

We conducted 8,676 (82.6%) interviews with PHC patients of the 10,500 intended. The South region presented the highest percentage of interviews (95.9%).

Table 1 presents the number of patients interviewed in PHC services, with weighted proportions and 95%CI according to demographic characteristics and by geographic region. The vast majority of respondents were women, 76% without differences between regions. Regarding age group, in the North and Midwest regions, the proportions of women aged from 18 to 39 and from 40 to 59 years were significantly higher than in the other regions. We highlight the distribution difference between ages of men and women, since the proportion of men aged 60 or over was higher than women of the same age group.

**Table 1:** Number and weighted proportion of patients interviewed in Primary Health Care services, according to demographic characteristics and geographic region. National Survey on Access, Use and Promotion of Rational Use of Medicines – Services, 2015.

Variable	North		Northeast		Midwest		Southeast		South		BRAZIL	
					
	%	95%CI	%	95%CI	%	95%CI	%	95%CI	%	95%CI	%	95%CI
Total number of respondents and weighted %												
	1,625 5.9		1,675 29.7		1,525 5.9		1,837 34.0		2,014 24.5		8,676 100.0	
No. of women interviewed												
	1.225		1.351		1.134		1.416		1.517		6.643	
	74.4	72.1–76.7	78.4	75.8–80.8	73.0	70.3–75.5	75.3	72.9–77.6	74.5	72.2–76.7	75.8	74.6–77.1
No. of men interviewed												
	400		324		391		421		497		2.033	
	25.6	23.3–27.9	21.6	19.2–24.2	27.0	24.5–29.7	24.7	22.4–27.1	25.5	23.3–27.8	24.2	22.9–25.4
Female sex per age group (years)												
18 to 39	59.9	57.0–62.8	45.6	42.3–49.0	51.0	47.6–54.5	39.5	36.5–42.6	38.4	35.6–41.3	42.9	41.3–44.6
40 to 59	27.6	25.1–30.4	35.3	32.2–38.6	31.8	28.7–35.1	39.0	36.0–42.0	40.4	37.5–43.4	37.1	35.6–38.8
60 and +	12.4	10.6–14.5	19.1	16.6–21.9	17.1	14.7–19.9	21.5	19.0–24.2	21.2	18.9–23.7	19.9	18.6–21.3
Male sex per age group (years)												
18 to 39	41.5	35.5–46.7	28.9	23.5–35.0	35.0	29.7–40.6	26.0	21.5–31.1	31.0	26.4–35.9	29.6	27.1–32.3
40 to 59	37.2	32.3–42.3	36.5	30.5–43.0	35.9	30.6–41.5	36.4	31.3–41.8	39.5	34.6–44.6	37.3	34.5–40.1
60 and +	21.3	17.4–25.9	34.5	28.7–40.9	29.2	24.3–34.6	37.6	32.4–43.1	29.5	25.1–34.3	33.1	30.4–36.0
Skin color												
White	15.2	13.5–17.2	25.7	23.2–28.4	29.1	26.5–31.8	41.3	38.7–44.0	63.1	60.6–65.5	39.7	38.3–41.1
Black	8.8	7.4–10.4	7.8	6.4–9.5	9.2	7.7–11.0	9.2	7.7–10.8	5.4	4.4–6.7	7.8	7.1–8.6
Yellow	0.1	0.0–0.3	0.3	0.1–0.8	0.6	0.3–1.3	3.8	2.8–5.0	0.2	0.1–0.7	1.5	1.1–1.9
Mixed race	75.1	72.8–77.3	66.0	63.1–68.7	60.7	57.8–63.5	45.2	42.5–47.9	30.4	28.1–32.8	50.5	49.0–51.9
Indigenous	0.7	0.4–1.3	0.2	0.0–0.7	0.3	0.1–0.9	0.2	0.1–0.7	0.1	0.0–0.4	0.2	0.1–0.4
Marital status												
Single	26.1	23.9–28.4	21.4	19.2–23.9	23.9	21.5–26.5	21.2	19.1–23.5	21.4	19.4–23.6	21.8	20.6–23.0
Married	39.0	36.5–41.5	45.7	42.8–48.7	49.0	46.1–51.9	52.0	49.3–54.7	49.8	47.3–52.4	48.6	47.2–50.1
Common–law marriage	26.4	24.1–28.7	21.3	19.0–23.8	15.1	13.1–17.3	12.1	10.4–14.0	12.8	11.2–14.6	16.0	15.0–17.1
Divorced/legally separated	4.0	3.1–5.1	5.3	4.1–6.7	6.7	5.4–8.3	7.8	6.4–9.3	7.6	6.4–9.1	6.7	6.0–7.5
Widower	4.5	3.5–5.7	6.2	4.9–7.8	5.1	3.9–6.5	6.8	5.5–8.2	8.2	6.9–9.7	6.7	6.0–7.5
Other	0.1	0.0–0.6	0.1	0.0–0.4	0.3	0.1–0.6	0.2	0.1–0.5	0.2	0.0–0.6	0.1	0.1–0.3

Source: PNAUM – Services, 2015.

In the self-reported skin color variable, the highest proportion was mixed race (50.5%), significantly higher in the North, Northeast, and Midwest, and lower in the South and Southeast regions. The proportion of black people was only lower than that of the Country (7.8%) in the South (5.4%), and the proportion of white people was significantly lower in the North, Northeast, and Midwest regions.

Most (64.6%) patients were married or had a common-law marriage, and this did not vary between regions; however, the proportion of common-law marriages in the North and Northeast was higher than other regions, and in the North the percentage of married people was significantly lower.

Some socioeconomic variables are presented in Table 2. The distribution of education levels between sexes and regions did not differ much, with a few exceptions: the proportion of illiterate patients was 10.3%, significantly higher in the Northeast and lower among women in the Midwest and in Southeast as a whole. We did not find differences in the proportions of elementary school and higher education between the regions and neither between sexes. Considering high school, women from the North region presented a higher percentage than in other locations.

**Table 2:** Socioeconomic characteristics of patients of Primary Health Care services, per region. National Survey on Access, Use and Promotion of Rational Use of Medicines – Services, 2015.

Variable	North		Northeast		Midwest		Southeast		South		BRAZIL	
					
	%	95%CI	%	95%CI	%	95%CI	%	95%CI	%	95%CI	%	95%CI
Education level per sex												
Illiterate												
F	7.9	6.4–9.7	12.5	10.4–14.9	5.8	4.4–7.7	7.2	5.7–9.0	11.2	9.4–13.2	9.7	8.8–10.8
M	13.6	10.4–17.6	18.1	13.6–23.5	7.4	4.9–11.1	8.5	5.8–12.1	11.0	8.1–14.6	11.9	10.1–14.0
Total	9.4	7.9–11.0	13.7	11.8–15.9	6.3	5.0–7.9	7.5	6.2–9.1	11.1	9.6–12.8	10.3	9.4–11.2
Elementary School												
F	48.5	45.6–51.5	53.9	50.6–57.2	54.2	50.8–57.5	52.1	49.0–55.1	49.0	46.0–52.0	51.8	50.2–53.5
M	60.6	55.4–65.4	58.5	52.1–64.6	66.8	61.2–71.9	63.2	57.8–68.3	54.6	49.5–59.6	59.8	56.9–62.6
Total	51.6	49.0–54.2	54.9	52.0–57.8	57.6	54.6–60.4	54.8	52.1–57.5	50.4	47.9–53.0	53.7	52.3–55.2
High School												
F	37.5	34.7–40.4	28.8	25.9–31.9	32.8	29.7–36.1	33.8	31.0–36.8	31.7	29.0–34.5	31.9	30.4–33.5
M	22.7	18.8–27.1	21.2	16.5–26.9	22.0	17.7–27.0	22.8	18.6–27.6	26.6	22.4–31.4	23.3	21.0–25.8
Total	33.7	31.4–36.2	27.2	24.6–29.9	29.9	27.3–32.6	31.1	28.7–33.6	30.4	28.1–32.8	29.9	28.6–31.2
Higher education												
F	6.0	4.8–7.6	4.8	3.5–6.4	7.2	5.6–9.1	6.9	5.5–8.7	8.2	6.7–9.9	6.5	5.8–7.4
M	3.2	1.9–5.3	2.3	1.0–4.8	3.8	2.2–6.7	5.6	3.6–8.5	7.8	5.5–11.0	5.0	3.9–6.3
Total	5.3	4.3–6.5	4.2	3.2–5.5	6.3	5.0–7.8	6.6	5.4–8.0	8.1	6.8–9.6	6.2	5.5–6.9
ABEP												
A+B	7.1	5.7–9.1	5.5	4.1–8.0	11.1	8.8–14.5	15.4	12.0–18.7	28.1	24.5–32.5	14.9	13.5–16.5
C	54.9	50.2–59.6	45.6	40.9–50.5	64.3	58.9–70.0	57.2	52.5–62.2	61.5	57.1–66.4	55.1	52.6–57.7
D+E	38.0	34.7–41.8	48.9	44.9–53.3	24.6	21.8–27.9	27.4	24.6–30.6	10.4	8.8–12.5	30.0	28.3–31.9
Government aid												
Bolsa Família benefit	36.5	30.3–43.1	46.7	41.0–52.5	19.0	15.3–23.4	14.4	11.5–17.7	11.3	9.2–13.8	24.8	21.7–28.2
Unemployment insurance	0.6	0.3–1.2	0.1	0.0–0.6	0.6	0.2–2.0	0.3	0.1–0.6	0.9	0.5–1.8	0.4	0.3–0.7
Does not have	58.8	53.5–63.9	47.2	43.4–51.1	77.0	73.6–80.1	82.1	78.3–85.3	74.6	68.1–80.2	68.2	64.2–72.0
Other	4.0	2.3–7.0	5.8	2.2–14.5	3.4	2.0–5.7	3.2	1.8–5.7	13.2	8.4–20.0	6.5	4.5–9.3
Health insurance												
Yes	2.5	1.9–3.3	8.5	6.0–12.1	6.2	5.0–7.6	9.6	8.0–11.5	16.7	14.7–18.8	9.8	8.9–10.7

ABEP: economic classification according to the Brazilian Association of Research Enterprises

F: female; M: male

Source: PNAUM – Services, 2015.

ABEP’s economic classification shows several regional differences between PHC patients. Classes A+B represented 14.9% and were significantly lower in the North and Northeast regions and much higher in the South. Slightly over half of respondents were classified as class C (55.1%). Classes D+E presented the highest proportions in the North and Northeast regions and lowest in the Midwest and South.

Another variable investigated was the receiving of government assistance. In Brazil, about 32% of respondents received some benefit. This proportion was higher in the North and Northeast region, and the benefit was *Bolsa Família*. Regarding unemployment insurance and other types of benefits, there were no differences between regions. Among PHC patients, 9.8% had health insurance. Only in the Southern region this proportion was significantly higher, while in the North and Midwest it was lower.

Most respondents (Table 3) replied that “never consumes alcohol” – 80.9% females and 61.6% males –, with significant difference between sexes, but no regional differences. The same pattern was observed between those who “drink less than once a month.” However, in patients who “drink once or more a month,” the prevalence in men was about three times higher than in women, and the South presented higher proportion than the other regions.

**Table 3:** Habits and lifestyle of Primary Health Care patients, according to sex and region. National Survey on Access, Use and Promotion of Rational Use of Medicines – Services, 2015.

Habits, lifestyle per sex	North		Northeast		Midwest		Southeast		South		BRAZIL	
					
	%	95%CI	%	95%CI	%	95%CI	%	95%CI	%	95%CI	%	95%CI
Alcoholic beverages consumption												
Never												
F	83.4	81.0–85.5	82.0	79.3–84.4	80.5	77.6–83.1	80.8	78.3–83.1	79.2	76.7–81.5	80.9	79.6–82.2
M	59.9	54.3–65.2	66.1	59.9–71.9	65.7	60.1–70.9	61.0	55.4–66.2	57.2	52.1–62.1	61.6	58.7–64.4
< once per month												
F	10.4	8.7–12.4	11.6	9.6–13.9	10.8	8.8–13.2	10.1	8.4–12.1	11.1	9.4–13.1	10.9	9.9–11.9
M	16.2	12.6–20.6	13.9	10.0–19.0	13.6	10.1–18.0	18.5	14.5–23.2	11.9	9.0–15.6	15.1	13.1–17.3
≥ once per month												
F	6.2	4.9–7.9	6.4	5.0–8.1	8.6	6.9–10.7	9.1	7.5–11.0	9.7	8.1–11.6	8.2	7.4–9.1
M	23.9	19.4–29.0	20.0	15.4–25.5	20.7	16.5–25.7	20.6	16.5–25.4	30.9	26.4–35.9	23.3	20.9–25.9
Currently smokes												
Yes												
F	6.2	4.8–7.9	8.3	6.6–10.3	10.3	8.3–12.0	12.0	10.1–14.2	15.5	13.5–17.7	11.3	10.3–12.4
M	19.4	15.4–24.3	17.0	12.7–22.4	18.7	14.6–23.5	20.1	16.0–25.0	22.5	18.5–27.1	19.8	17.5–22.2
Diets to lose weight												
Yes												
F	22.7	20.2–25.3	15.6	13.3–18.1	16.2	13.9–19.0	22.1	19.6–24.8	24.9	22.5–27.6	20.5	19.2–21.8
M	11.5	8.5–15.4	7.3	4.7–11.3	10.8	7.7–14.9	11.6	8.5–15.8	17.7	14.2–21.9	12.0	10.2–14.0
Avoids salt intake												
Yes												
F	66.7	63.7–69.5	58.4	55.1–61.7	56.1	52.7–59.5	58.1	55.0–61.1	67.7	64.8–70.4	60.9	59.2–62.5
M	54.7	49.1–60.2	53.4	47.0–59.7	49.4	43.7–55.2	49.5	44.0–55.0	60.3	55.2–65.2	53.7	50.7–56.6
Reduces fat intake												
Yes												
F	58.4	55.4–61.4	57.1	53.0–60.4	53.7	50.2–57.1	53.3	50.1–56.4	65.4	62.6–68.2	57.7	56.1–59.3
M	48.9	43.3–54.4	46.6	40.3–53.0	37.2	31.9–42.9	41.6	36.3–47.2	49.3	44.2–54.4	45.1	42.2–48.0
Reduces sugar intake												
Yes												
F	50.6	47.6–53.7	44.1	40.8–47.4	42.0	38.7–45.5	43.4	40.4–46.5	52.8	49.8–55.7	46.2	44.6–47.9
M	41.9	36.5–47.5	33.9	28.2–40.1	33.0	27.9–38.6	35.7	30.5–41.1	42.3	37.4–47.4	37.1	34.3–40.0
Uses sweetener												
Yes												
F	17.8	15.6–20.2	20.6	18.0–23.4	12.1	10.0–14.5	22.4	19.9–25.1	21.6	19.3–24.1	20.8	19.5–22.2
M	16.1	12.5–20.6	22.8	17.9–28.6	9.9	7.0–13.8	19.5	15.4–24.2	19.0	15.3–23.2	19.4	17.2–21.9
Performed physical exercises in the past 3 months												
Yes												
F	22.1	19.4–25.0	18.9	16.6–21.3	20.5	18.0–23.4	21.7	19.3–24.2	38.6	35.7–41.5	25.3	23.9–26.8
M	24.0	19.6–29.0	22.9	18.0–28.6	26.5	21.8–31.9	20.5	16.4–25.2	46.4	41.3–51.5	28.5	26.0–31.2

F: female; M: male

Source: PNAUM – Services, 2015.

We observed higher prevalence of smoking among men, without differences by region. Women showed a higher rate than men among those who diet to lose weight. Women of the South region have higher proportion, and those from Northeast and Midwest regions have lower proportion. Among men, the highest percentage was also in the South.

Concerning avoiding salt intake, women showed higher rates than men, being higher in the South and North regions. Among men, we found no regional differences of this intake. The reduction of fat in the diet presented a similar distribution to that of the salt: higher proportion of women, especially in the South, and no differences between men. Women also reported decrease in the ingested sugar, especially in the South. Among men, this prevalence was lower, without regional differences. Regarding the use of sweetener, we found no difference between sexes, but we verified a lower proportion in the Midwest region for both sexes. Regarding the question about performing some physical exercise or practicing some sport in the past three months, we observed no difference between sexes.

Table 4 shows that slightly over half of PHC patients evaluated their health as very good and good, except for the Northeast residents, who had the highest percentage of answers in the category neither bad nor good (42.1%). We verified no differences between regions in the proportion of very bad and bad, 7.9%.

**Table 4:** Health conditions reported by Primary Health Care patients. National Survey on Access, Use and Promotion of Rational Use of Medicines – Services, 2015.

Health condition	North		Northeast		Midwest		Southeast		South		Brazil	
					
	%	95%CI	%	95%CI	%	95%CI	%	95%CI	%	95%CI	%	95%CI
Health self–assessment												
Very good and good	59.7	55.9–63.8	47.5	43.5–51.9	58.6	53.0–63.7	62.1	57.7–66.8	60.4	56.4–64.5	57.0	54.7–59.3
Neither bad nor good	32.3	29.8–34.8	42.1	39.2–45.1	34.2	31.4–37.0	32.1	29.7–34.7	31.0	28.7–33.5	35.0	33.6–36.4
Very bad and bad	7.9	6.3–10.1	10.2	8.0–12.8	7.2	5.3–9.6	5.6	4.2–7.5	8.5	6.7–10.8	7.9	7.0–9.0
Chronic disease/condition diagnosed by a doctor or other health professional												
Hypertension	25.6	23.3–28.0	37.0	34.2–39.9	32.4	29.7–35.2	41.6	38.9–44.3	40.6	38.1–43.1	38.6	37.1–40.0
Dyslipidemia	19.2	17.2–21.3	22.8	20.4–25.4	18.6	16.4–21.0	21.6	19.5–24.0	25.7	23.6–28.0	22.7	21.5–23.9
Arthritis/Rheumatism	14.0	12.3–16.0	16.7	14.7–19.0	18.9	16.7–21.4	30.9	27.8–34.2	26.4	24.2–28.8	19.4	18.3–20.6
Depression	8.5	7.1–10.0	15.2	13.2–17.5	13.0	11.2–15.1	16.8	14.9–18.9	28.3	23.6–30.7	18.5	17.4–19.6
Diabetes	9.9	8.5–11.7	11.6	9.8–13.6	10.3	8.6–12.2	15.8	13.9–17.9	14.6	12.9–16.4	13.6	12.6–14.6
Chronic Lung Disease	8.4	7.1–10.0	7.3	5.9–8.9	8.6	7.1–10.4	8.8	7.4–10.5	13.9	12.2–15.7	9.6	8.8–10.4
Heart disease	3.5	2.6–4.6	5.7	4.5–7.2	6.5	5.2–8.1	7.8	6.4–9.4	11.1	9.6–12.8	7.7	6.9–8.5
Cerebrovascular accident	1.5	1.0–2.2	3.3	2.4–4.5	1.1	0.6–1.8	1.3	0.8–2.1	3.6	2.7–4.6	2.5	2.1–3.0

Source: PNAUM – Services, 2015.

Among the chronic diseases/conditions diagnosed by a physician or other health care professional, reported by the respondents, hypertension was the most prevalent (38.6%), followed by dyslipidemia (22.7%), lower in the North and Midwest regions. Arthrosis, arthritis, or rheumatism (19.4%) showed differences between the regions, with higher proportions in the Southeast and South regions and lower ones in the North. The diagnosis of depression had higher prevalence in the South (28.3%), and lower proportion in the North and Midwest. The lowest prevalence of diabetes was found in the North and Midwest regions.

Chronic pulmonary disease presented higher prevalence in the South, as well as heart diseases, which were only lower in the North. The proportion of Cerebrovascular Accident (CVA) in the Country was 2.5%, with lower prevalence in the Midwest.

Most respondents (73%) always sought for care in PHC units, but in smaller proportion in the North and Midwest regions. In these two regions, answers in the category “sometimes” were more frequent than in the other regions, while “rarely” was significantly higher in the Midwest than in the rest of the Country.

Regarding the emergency care in past year, 22.9% of patients used this service, with a much higher demand in the South and a lower one in the Southeast. Hospitalization in the past 12 months accounted for 9.6%, without differences between regions of the Country.

When asked about the use of medicines in the last month, 75.6% answered affirmatively, though with a higher proportion in the South and a lower one in the North and Midwest regions. Concerning the place where they sought for medicines in SUS pharmacies, 43.0% answered that they only sought in the UBS, with higher proportion of patients in the South and lower in the Northeast and Midwest regions. Among all respondents, 60.3% of PHC patients demanded only SUS pharmacies.

Those who have not sought for any medicine in the past three months (11.6%) presented regional differences: higher percentages in the North and South and lower ones in the Northeast region. Many patients (21.4%) answered “I did not use any SUS pharmacy,” without differences between regions.

## DISCUSSION

The main features of the PHC patients with more than 17 years of age are: young women and older men, of mixed race, married or in a common-law marriage, more than half with elementary school, social classification C, without health insurance, and low consumption of alcohol. The self-assessment of health condition rated as good and very good was reported in a lower proportion when compared with the general population, while there was higher prevalence of chronic diseases such as hypertension, diabetes, and depression, and most patients depend on SUS to get medicines. The South region is where the prevalence both of diseases and risk factors is higher, which is opposite of the situation in North.

The interview addressed several aspects of patients of PHC services. The originality of our research and its national scope hinders the comparison of our results with official data or other articles. The study used different criteria in the data collection on risk factors, such as alcoholism.

We could only establish the statistical significance between the variables by the use of the confidence interval due to the weighting of results according to the size of the city and geographical regions.

The optimal parameters of data comparison of PHC patients of this research would be those of SIAB, but few of them are fit to this purpose, for involving different age groups and criteria for defining diseases or risk factors, in addition to the lack of variables and questions related to the development of the system in the thousands of Brazilian cities. Besides, people registered in SIAB not necessarily attend PHC services.

Many studies on PHC have characterized their patients, but in a more restricted way, focusing on specific aspects of a health problem or even a management problem. Hence, we used data from the *Sistema de Vigilância de Fatores de Risco e Proteção para Doenças Crônicas por Inquérito Telefônico* (Vigitel – Surveillance System of Risk and Protection Factors for Chronic Diseases by Telephone Survey) 2014^14^ and from the *Pesquisa Nacional de Saúde* (PNS – National Survey on Health) 2013^6^, considering that both surveys have different methodologies, which enable estimates for the Brazilian population, unlike the interviewees of our study, who comprise a representative sample of PHC patients circumscribed to regions of the Country.

Our study indicates that most patients are women, as already observed in monographies^17^ and in national^4^ ^,^ ^9^ ^,^ ^16^ and international^18^ ^,^ ^19^ ^,^ ^22^ ^,^ ^23^ studies.

Although men have higher rates of morbimortality, they resist more to seek primary health care, which was one of the reasons for the creation of the *Política Nacional de Atenção Integral à Saúde do Homem* (PNAISH – National Policy of Integral Care for Men’s Health) by the Brazilian Ministry of Health^13^. The fact that the proportion of men aged 60 years or over is higher than that of women of the same age group (33% compared with 20%) reinforces the observation that men seek health care when older and probably only when they have symptoms^13^.

In a study that analyzed patients of SUS and other who did not attend SUS services^20^, white people constituted 48.1% of SUS patients, a higher proportion than the observed in our study between PHC patients (39.7%); therefore, the presence of mixed race and black people was higher: 58.3% among PHC patients and 51.9% in all SUS. The proportion of mixed race and black people is similar to that found on the last census^5^.

About 64% of patients had a spouse, without differences between regions. In Belo Horizonte, the percentage in regular patients was lower, 52%^7^.

Regarding education level, in our study 54% of patients had elementary school, a similar percentage to the one found in Belo Horizonte^7^.

Most patients (55%) of PHC services are from the social class C of ABEP. This same value was observed in the household survey of PNAUM. However, 30% of patients are from the D+E class, and this percentage in the survey was 22%^2^.

According to the *Agência Nacional de Saúde Suplementar* (ANS – National Regulatory Agency for Private Health Insurance and Plans), in June 2016, 48.5 million people had health insurance with or without dental care, corresponding to about 25%^3^ of the Brazilian population. As expected, the population of PHC patients presented a lower coverage, accounting for 10%. The lowest difference between the general population and that of PHC services was observed in the South region, 25% and 17% respectively.

Regarding alcohol consumption, we observed that 8% of women patients of PHC consume one or more shots per month, against 23% of men. According to Vigitel 2014 data^14^, the prevalence of excessive alcohol consumption in the Country (more than four shots for women and more than five shots for men on the same occasion in the past 30 days) is 9.4% in the female sex and 12.8% in the male sex. Among PHC patients, alcohol consumption was observed once or more per month, as well as in the PNS 2013^6^, whose prevalence in women was similar to that of PNAUM, although much higher in men. In addition, lower proportions were found in the North and South regions in both sexes. In SIAB, the percentage of alcoholism in people older than 15 years of age (December 2015) was 0.5%^b^.

In all surveys, the prevalence of smoking was higher in men than in women. The rates of women in PNS 2013^6^ and of PHC patients are similar, a bit lower than those of Vigitel 2014^14^. Among men, these prevalences vary significantly: they are higher in our study, 19.8%; in PNS 2013^6^, 15.9%, and in Vigitel 2014^14^,12.8%. In a recent study^9^ conducted in Belo Horizonte with PHC patients, the prevalence of smokers was 15.8%, similar to that of PNS 2013^12^, with higher proportion between men. In Pelotas^8^, this percentage was 23.4%, the same value found in Spain^18^.

If a reasonable portion of PHC patients reports dieting to lose weight (higher in the South), these percentages are not necessarily related to the frequency of overweight (BMI ≥ 25), which, according to Vigitel 2014^14^, was 49.1% between women and 56.5% between men in the Country, with statistically significant difference between sexes.

In Vigitel 2014^14^ survey, salt intake between men was higher than between women, and Porto Alegre was the capital with higher intake between women, and Florianópolis, between men. Among patients of our study, we observed that women avoided salt intake in a higher proportion than men, mostly in the South and North regions.

The highest frequency of PHC patients who avoided fat intake was observed in the South and between women, and, according to Vigitel 2014^14^, they consume fatty meat in a lower proportion (21.7%) than men (38.4%). However, there is no measure per region, only capitals.

The sweet food consumption estimated by PNS 2013^6^ was 21%, without differences between sexes, but lower in the North and higher in the South. In Vigitel 2014^14^, women’s consumption (20.3%) was much higher than men’s (15.6%), with the highest percentages in Porto Alegre and Curitiba. It is noteworthy that in the South there is a higher proportion of PHC patients who reported to decrease the amount of sugar in the diet.

The criteria of physical exercises in PNAUM – Services are different from Vigitel 2014^14^, in which there are several measures, among them physical activity in spare time for 150 minutes of moderate activity per week, 30.0% in women and 41.6% in men, with statistically significant difference, and Florianópolis with the highest percentage of all the capitals of the Country, 47.1%, and São Paulo, the lowest, 30.4%. The overall inactivity rate is 15.4%, without differences between sexes. The differences between female and male PHC patients were not significant, being 25% and 29%, respectively. No significant difference was observed between regions either. The criterion used does not necessarily indicate physical inactivity for those who declared not exercising regularly over the past three months.

Regarding health conditions reported in Vigitel 2014^14^, only the negative self-assessment of health was found (4.4%), ranging from 3.1% in Campo Grande to 7.9% in Manaus (the only city with a significantly higher percentage), whose rate was similar to that found among PHC users in the North region and in the entire Country (Table 4). However, in PNS 2013^6^, the good and very good self-assessment of health was 66.1% for Brazil, ranging from 56.7% in the Northeast to 71.5% in the Southeast, rates higher than those of PHC patients. In patients of a family physician in Spain^19^, 9% considered their health as bad or very bad and 78% as good and very good. In regular patients of the *Estratégia Saúde da Família* (ESF – Family Health Strategy) of Belo Horizonte^13^, 4% considered their health as bad or very bad. This value was similar to that observed in PHC patients of the Southeast region.

The prevalence of diseases diagnosed by physicians or other health care professional among PHC patients was higher than those mentioned in PNS 2013^6^ and Vigitel 2014^14^. This was already expected, because the interviewed patients were in the units by some health condition or to get medicines for themselves or family members.

The percentage of hypertension in people with 15 years of age or over was: 9% according to SIAB, in December 2015; among those over 17 years old, in PNS 2013^6^, 21%; in Vigitel 2014^14^, 25%; in PNAUM – Household Survey, 24%; and in PNAUM – Services, 39%. In the survey among patients of Family Health teams, in the northeast of Minas Gerais^21^, the main problem was related to circulatory system diseases, basically arterial hypertension. In Portugal^19^, arterial hypertension (non-aggravated) accounted for 6.4%.

High cholesterol, according to Vigitel 2014^14^, was 20%, similar to our study (23%). In PNS 2013^6^, it was lower, 13%. Also in PNS 2013^6^, arthritis or rheumatism showed prevalence of 6.4%, a rate far lower than that of PHC patients, without differences between regions. Depression, according to PNS 2013^6^, affected 7.6% of the Brazilian population, with prevalence significantly lower in the North and Northeast and higher in the South, similar to that of PHC patients, but at lower levels. In Portugal, the prevalence of depression was 11%^19^, lower than the percentage of our study (18.5%).

Diabetes reported in Vigitel 2014^14^ was 8.0%, accounting for 6.5% in PNS 2013^6^, with lower rates in the North and Midwest regions and lower than those of PHC patients (13%). In SIAB, the percentage of diabetics registered in December 2015 was 2.6%.

Other diseases researched in PNS 2013^6^ were: asthma (affecting 4.4% of the population), with lower frequency only in the Northeast; heart disease (4.2%), with higher prevalence in the South and lower in the North and Northeast; and CVA (1.5%), with no regional differences. In this study, all prevalences for these diseases were higher.

Table 5 shows that the surveys conducted on demand of health services and medicines, including PNS 2013^6^, used criteria and periods that differ from our study, except concerning hospitalization in the past 12 months of the general population, which was 6.0%, lower prevalence than that among PHC patients (9.6%). This significant difference could be explained by the characteristics of high vulnerability found in this population, with higher rates of morbidity for the surveyed chronic diseases/conditions and, probably, because they seek or are met in PHC services when these diseases are in more advanced stages.

**Table 5:** Search for health services and medicines, per region. National Survey on Access, Use and Promotion of Rational Use of Medicines – Services, 2015.

Search for service and medicines	North		Northeast		Midwest		Southeast		South		Brazil	
					
	%	95%CI	%	95%CI	%	95%CI	%	95%CI	%	95%CI	%	95%CI
Search for care at PHCU												
Always	67.0	64.4–69.4	75.6	73.0–78.1	60.4	37.5–63.3	73.5	71.0–75.8	73.4	71.0–75.6	73.0	71.7–74.2
Repeatedly	4.6	3.6–5.9	3.1	2.2–4.3	6.3	4.9–7.9	5.2	4.2–6.6	1.8	1.2–2.6	3.8	3.3–4.4
Sometimes	19.2	17.1–21.4	13.0	11.1–15.1	19.4	17.1–21.8	11.9	10.2–13.7	16.5	14.7–18.6	14.2	13.2–15.2
Rarely	6.0	4.8–7.3	5.2	4.0–6.7	10.6	8.9–12.6	7.1	5.8–8.6	5.2	4.1–6.5	6.2	5.5–6.9
Never	0.0	0.0–0.3	0.0	0.0–0.1	0.2	0.1–0.7	0.4	0.2–0.9	0.1	0.0–0.5	0.2	0.1–0.4
First time	3.2	2.4–4.4	3.0	2.2–4.2	3.1	2.3–4.3	1.9	1.4–2.8	3.0	2.3–4.0	2.7	2.3–3.2
Used emergency services in the past year												
Yes	21.0	19.0–23.3	22.3	20.0–24.9	26.1	23.3–28.8	18.3	16.3–20.5	29.5	27.2–31.9	22.9	21.7–24.1
Hospitalization in the past 12 months												
Yes	8.8	7.4–10.5	9.4	7.8–11.3	12.5	10.0–14.6	7.6	6.3–9.1	12.1	10.5–13.9	9.6	8.8–10.5
Took medicine in the past month												
Yes	64.5	62.0–67.0	73.7	71.1–76.2	70.7	68.0–73.3	74.5	72.1–76.8	83.4	81.4–85.2	75.6	74.4–76.9
Sought medicines in SUS pharmacies in the past 3 months?												
Only in UBS	44.9	42.3–47.6	38.3	35.4–41.2	33.7	31.1–36.6	43.2	40.5–45.9	50.1	47.5–52.6	43.0	41.5–44.4
Only in other SUS Pharmacies	4.2	3.3–5.4	4.5	3.4–6.0	9.6	8.0–11.4	5.5	4.4–6.9	6.7	5.5–8.1	5.7	5.1–6.4
In both	18.4	16.4–20.6	16.7	14.6–19.0	21.4	19.0–23.9	19.8	17.7–22.1	16.6	14.8–18.6	18.1	17.0–19.2
Did not seek	16.0	14.2–18.0	6.5	5.2–8.1	9.6	8.1–11.3	12.8	11.1–14.8	15.5	13.7–17.5	11.6	10.7–12.5
Did not use any SUS pharmacy	16.5	14.5–18.6	33.0	30.9–36.5	25.6	23.1–28.3	18.1	16.1–20.2	11.1	9.6–12.8	21.4	20.2–22.6

UBS: basic health unit; SUS: Brazilian Unified Health System

Source: PNAUM – Services, 2015.

Regarding hospitalization in the past 12 months, in the study conducted in Belo Horizonte^7^, the proportion of hospitalizations among patients of traditional UBS was 3.1%, and 3.6% in the ESF, without differences between these care units, but lower than that of our study (10%). However, in PNS 2013^6^, 6% of the whole Country reported hospitalization in the past 12 months, with South and Midwest regions presenting higher rates than the Brazilian average, similar to the prevalence observed among PHC patients, but with slightly higher percentages.

The use of medicines in the past month, in a study performed with PHC patients of Lorena, São Paulo, in 2005, accounted for 51.3%^4^, a lower proportion than that of our study, conducted ten years later (75.6%).

The limitation of our research is that the interviews were conducted only with the patients who were present in PHC services, whose characteristics are personal and different of the general population and of patients of other services. The prevalence of diseases and use of medicines may have been overestimated. The sampling process excludes patients who require medicines in short supply at the time this survey was conducted. However, the results may contribute to a greater understanding of the characteristics of PHC patients in Brazil and may serve as a reference for further studies.

## Footnotes

a Ministério da Saúde (BR). Portal da Saúde. Sobre o Departamento de Assistência Farmacêutica – DAF. Brasília (DF); [s.d.] [cited 2016 Dec 8]. Available from: http://portalsaude.saude.gov.br/index.php/oministerio/principal/secretarias/sctie/daf/componente-basico-da-assistencia-farmaceutica

b Ministério da Saúde (BR), DATASUS. Sistema de Informação e Atenção Básica: cadastramento familiar – Brasil. Brasília (DF) [cited 2017 Jan 31]. Available from: http://tabnet.datasus.gov.br/cgi/deftohtm.exe?siab/cnv/SIABFbr.def

c Associação Brasileira de Empresas de Pesquisa. Critério de classificação econômica Brasil: alterações na aplicação do Critério Brasil, válida a partir de 01/01/2014. São Paulo: ABEP; 2014 [cited 2016 Mar 1]. Available from: www.abep.org/Servicos/Download.aspx?id=01
